# Age-Related Differences With Immersive and Non-immersive Virtual Reality in Memory Assessment

**DOI:** 10.3389/fpsyg.2019.01330

**Published:** 2019-06-11

**Authors:** Adéla Plechatá, Václav Sahula, Dan Fayette, Iveta Fajnerová

**Affiliations:** ^1^National Institute of Mental Health, Klecany, Czechia; ^2^Third Faculty of Medicine, Charles University, Prague, Czechia

**Keywords:** virtual reality, memory assessment, aging, immersion, neurocognitive methods

## Abstract

Memory decline associated with physiological aging and age-related neurological disorders has a direct impact on quality of life for seniors. With demographic aging, the assessment of cognitive functions is gaining importance, as early diagnosis can lead to more effective cognitive interventions. In comparison to classic paper-and-pencil approaches, virtual reality (VR) could offer an ecologically valid environment for assessment and remediation of cognitive deficits. Despite the rapid development and application of new technologies, the results of studies aimed at the role of VR immersion in assessing cognitive performance and the use of VR in aging populations are often ambiguous. VR can be presented in a less immersive form, with a desktop platform, or with more advanced technologies like head-mounted displays (HMDs). Both these VR platforms are associated with certain advantages and disadvantages. In this study, we investigated age-related differences related to the use of desktop and HMD platforms during memory assessment using an intra-subject design. Groups of seniors (*N* = 36) and young adults (*N* = 25) completed a virtual Supermarket Shopping task using desktop and HMD platforms in a counterbalanced order. Our results show that the senior performances were superior when using the non-immersive desktop platform. The ability to recall a shopping list in the young adult group remained stable regardless of the platform used. With the HMD platform, the performance of the subjects of both groups seemed to be more influenced by fatigue. The evaluated user experiences did not differ between the two platforms, and only minimal and rare side effects were reported by seniors. This implies that highly immersive technology has good acceptance among aging adults. These findings might have implications for the further use of HMD in cognitive assessment and remediation.

## Introduction

Cognitive functions play an important role in our everyday lives, governing our thoughts and actions and enabling successful adaptation to changes occurring in the surrounding environment (Sternberg et al., 2012). Our cognitive abilities can be affected during aging by common physiological processes and by neuropsychiatric and neurological disorders such as Alzheimer’s disease (AD) and vascular impairments. In the context of demographic aging, with adults over 65 years of age forming 15% of the entire United States population (United States Census Bureau, 2018) and 19.2% of the European Union population (Eurostat, 2018) the problems associated with older age are gaining in importance. Physiological aging typically accompanies decline across all cognitive domains, mainly in processing speed, divided attention, language, visuospatial abilities, memory, and executive functions (Harada et al., 2013). The most robust manifestation of physiological aging is visible memory decline (Rönnlund et al., 2005); this is subjectively the most relevant for seniors (Harada et al., 2013). In AD diagnostics, episodic memory plays an important role. The deficit in episodic memory in seniors is strongly pronounced and can be demonstrated both in errors of recent autobiographical memory and laboratory assessments using recall and recognition tasks (Rönnlund et al., 2005). The deficit in episodic memory is detectable using neuropsychological measurements up to 10 years before the diagnosis of AD; it could therefore possibly be used as a marker for early diagnosis (Bäckman et al., 2001; Boraxbekk et al., 2015). Early diagnosis can result in better-timed and more effective interventions, which might delay further progression of the cognitive decline (Naqvi et al., 2013). Thus, in the light of increasing life expectancy, the assessment of age-related memory changes is growing in relevance.

Memory deficit is usually assessed using classic paper-and-pencil neuropsychological methods; such methods have been questioned for their lack of ecological validity since 1978 (Neisser, 1978). Ecological validity can be understood as the degree to which experimental conditions approximate conditions in the real-world environment (Tupper and Cicerone, 1990) or the extent to which the test performance or study results can be generalized to real-life settings (Franzen, 1997). Classic neuropsychological tests fail to resemble real-world demands, and there has been increasing interest in neuroscience in the use of advanced technology (Parsons, 2015). Computer technologies enable precise test administration, stimulus presentation, and automatic response recording. Virtual reality (VR) is gaining in popularity due to its ability to present three-dimensional objects and create complex virtual environments (VE) that might be realistic and ecologically valid while also being precisely controllable (Parsons, 2015).

Important term linked to VR is immersion. Immersion was defined by Slater (2009) as a characteristic of the technology used for VE presentation; basically, the higher the quality of the system, the higher the level of immersion (for example, in terms of the tracking latency, the size of the field of view, or the visual quality of the scene and images). Immersion is also determined by the ability of the system to support sensorimotor contingencies, such as how the technology responds to the action performed by the user to perceive reality, e.g., turning the head to change the gaze direction (O’Regan and Noë, 2001).

Despite the obvious benefits of HMD technology (multisensory stimulation, tracking of the head and body movements, higher sense of presence), results of previous studies are not conclusive in terms of the advantages of HMD in assessing cognitive performance nor in its usability in the senior population. Previous studies have shown superior performance either using HMD (Bowman et al., 2009; Murcia-López and Steed, 2016) or using less immersive technology, such as desktop or large screen platforms (Ruddle et al., 1999; Mania and Chalmers, 2001; Sousa Santos et al., 2009). Moreover, the majority of the studies comparing HMD and less immersive technologies in terms of cognitive performance have focused on navigation or spatial memory (Ruddle et al., 1999; Bowman et al., 2009; Sousa Santos et al., 2009; Murcia-López and Steed, 2016); few studies have investigated other cognitive domains (Mania and Chalmers, 2001; Rand et al., 2005). The findings considering preference and usability of HMD seem to be more consistent, showing a preference for higher immersion technologies, mainly in terms of increased motivation (e.g., Moreno and Mayer, 2004; Richards and Taylor, 2015; Parong and Mayer, 2018), more intuitive action control, and greater enjoyment associated with task fulfillment (e.g., Sousa Santos et al., 2009). Most of these studies (except Rand et al., 2005) were conducted on young subjects; their findings cannot be easily generalized to the senior population. There is not enough evidence indicating the applicability and acceptance of HMD for cognitive assessment and training in seniors.

The aims of our study are:

• To evaluate the possible effects of immersion level on episodic memory performance for diagnostic purposes;
• To evaluate user experiences of immersive and non-immersive technology across different age groups; and
• To test the validity of a memory task designed in a complex ecologically valid virtual environment in young adults and seniors in terms of the applied immersion level.

We used an intra-subject design to investigate the role of the level of immersion on performance and user experience in memory assessment. We were interested in the difference in acceptance as evaluated by seniors (60 years and older) and by young adults (up to 40 years old). HMD has been previously considered more intuitive and motivating (Martínez-Arán et al., 2004; Richards and Taylor, 2015; Parong and Mayer, 2018). We therefore hypothesized that the platform used will affect user experience. We expected to find differences between platforms in memory performances, as the more immersive technology is seen as more engaging and thus might result in better cognitive outcomes. This hypothesis is in contrast with some previous findings that associate the HMD platform with lower cognitive performance. We speculate that recent innovations in the technology of virtual glasses might lead to a different outcome.

## Materials and Methods

### Participants

**FIGURE 1:**
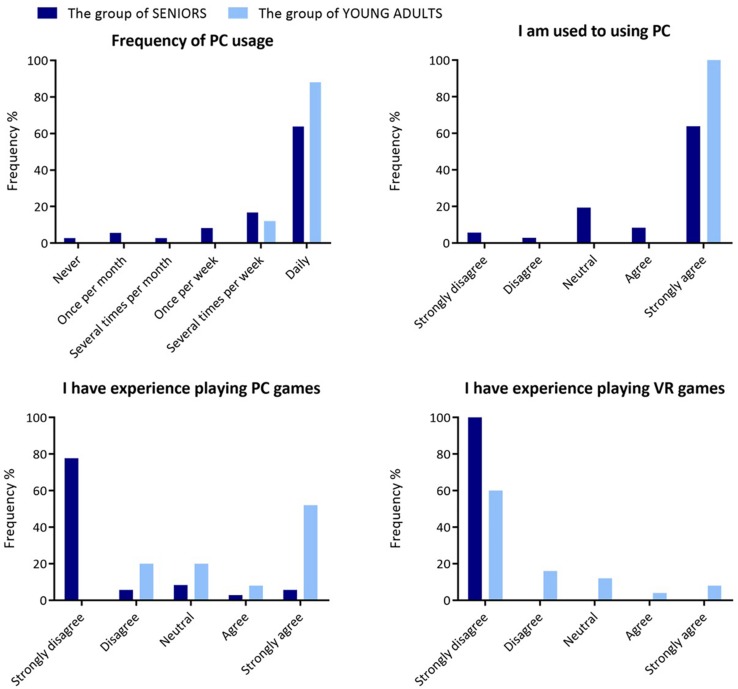
Distribution of group characteristics related to their experience with computers and virtual reality. The graphs show the frequency of the answers to the specific statements from the usability questionnaire part I (see Table 2).

Thirty-six seniors (13 males and 23 females, mean age = 69.47; SD = 7.39; age range = 60–91) and 25 young adults (9 males and 16 females, mean age = 25.4; SD = 5.13; age range = 19–39) voluntarily participated in this study. All participants signed an informed consent form containing information about the experiment procedure and exclusion criteria. The study was approved by the ethics committee of the NIMH in Klecany. Seniors were recruited from the database of the Department of Cognitive Disorders (NIMH) where they were neuropsychologically evaluated and classified as cognitively healthy. Young adults were recruited from the NIMH database of healthy volunteers to be matched in sex and education level to the group of seniors. Participants were not included in the study if they had major neurological disorders, diagnosed psychiatric illness, recent traumatic brain injury, brain surgery, or another illness involving major visual or movement impairment that would prevent them from participating in the experiment. The groups did not differ in demographic characteristics (apart from age). Detailed characteristics of the groups of seniors and young adults are presented in Table 1. Figure 1 presents group-specific distributions of characteristics related to the computer/videogame experience obtained from the usability questionnaire (see section “Usability Questionnaire”).

**TABLE 1:** Summary table of demographic characteristics for individual age groups.

		**Group of seniors (*N* = 36)**	**Group of young adults (*N* = 25)**	**Group difference**	
**Demographic**		**Mean score (SD)**		**Mann-Whitney U**	***p***
Age		69.47 (7.39)	25.40 (5.13)		
		**Frequency (%)**			
Sex	Males	13 (36.1%)	9 (36%)		
	Females	23 (63.9%)	16 (64%)		
Level of education	Vocational school	3 (8.3%)	0 (0%)		
	High school	15 (41.7%)	13 (52%)		
	University degree	18 (50%)	12 (48%)		
Education (Years)		15.89 (3.86)	17.24 (3.8)	−1.353	0.181

### Cognitive Evaluation

All participants were assessed using standard neuropsychological methods to briefly evaluate their cognitive performance, particularly learning and declarative memory, psychomotor speed, and mental flexibility.

The Czech version of the Rey Auditory Verbal Learning Test (RAVLT) (Rey, 1964; Preiss, 1999) was used as a standard measure of episodic memory (Pause et al., 2013) evaluating verbal learning and delayed recall. For the group comparison we used the total number of recalled words (RAVLT I-V) and the number of words correctly recalled after a 30-min delay (RAVLT delayed).

The Czech version of the Trail Making Test (TMT) (Reitan and Wolfson, 1985; Preiss and Preiss, 2006) was used as a standard measure of psychomotor speed and attention. Part A (TMT-A) evaluates psychomotor speed and visual attention; part B (TMT-B) is focused on visuospatial working memory and mental flexibility.

### The Virtual Supermarket Shopping Task

The virtual Supermarket Shopping Task (vSST) was specifically designed using Unity Engine software^1^ for assessing episodic memory in an ecologically valid environment. The desktop version of the task was tested on patients with chronic schizophrenia and on healthy young adults (Plechatá, 2017; Plechatá et al., 2017). Other than feasibility testing in a pilot study using both desktop and HMD platforms, no sample of seniors has previously been assessed using the vSST task. The task was originally created in order to assess everyday functioning in a virtual environment that reflects real-world situations. The task is similar to neuropsychological multiple errand tasks, but it is performed in virtual reality, which ensures a safe environment and complete control over the presented stimuli (Parsons, 2015). A similar fully immersive shopping task was recently validated as a measure of episodic memory performance (Corriveau Lecavalier et al., 2018).

The virtual environment of the vSST resembles a grocery store in which the subject is supposed to remember a shopping list and later find and collect recalled items in the virtual shop. Prior to the beginning of the testing, the participant has time to explore the VE and to become familiar with the control system. The length of the *exploration phase* differed according to the platform used (10 min for HMD and 4 min for desktop). Each trial of the vSST task consist of two phases: the *acquisition phase* (presentation of the shopping list) and the *recall phase* (testing the recall of the shopping list by direct collection of individual items in the virtual supermarket). Between the acquisition and recall phases, participants were instructed to play a visuospatial game, the LEU Brain Stimulator^2^, for 3 min as a distraction task. The length of the delay was directly controlled by the vSST application, and the countdown was displayed on the screen.

The vSST had four consecutive levels of increasing difficulty (requiring remembering three, five, seven, and nine items on the shopping list). The first trial, with three items, was meant as a pretraining trial and its results were not further analyzed. The length of the acquisition phase increased automatically by 5 s for each item added to the list (i.e., 15 s for three items; 25 s for five items; 35 s for seven items; 45 s for nine items). After completing each recall phase, the results (number of errors, trial time, and trajectory) were presented to the participant. The beginning of the next acquisition phase was controlled by the participant, who could start off the next trial by pressing a confirmation button with the mouse or with the HTC VIVE controller.

In order to allow for repeated assessment using the vSST, two task variants of the shopping list were created for each difficulty level (variant A and variant B). Both variants were demonstrated to be comparable in terms of difficulty in the previous study (Plechatá, 2017).

The vSST makes it possible to evaluate three main variables: errors (omissions – missing items, and intrusions – additional items) committed while recalling individual items from the shopping list, time spent solving the task (recalling and picking up the item) and trajectory length (distance traveled in VE). For the purposes of this study, we report only the number of errors directly related to memory recall. Moreover, the movement control was different across the platforms (teleportation in HMD together with free real-world movements vs. walking using a keyboard in the desktop platform); therefore, platforms are not fully comparable in terms of trajectory traveled and solving time.

### Usability Questionnaire

For this study, we developed a 55-item usability questionnaire inspired by previous usability studies (Lewis, 1995; Kaufmann and Dünser, 2007). The questionnaire has four main parts, which are summarized in Table 2. Responses considering user experience with platforms and comparison of the platforms were recorded using a five-point Likert scale (ranging from “strongly disagree” designated as 1 to “strongly agree” designated as 5). In the analysis of the questionnaire, we worked with cumulative raw scores for each platform. The cumulative score was computed by combining the score of 14 items. From the UQ II HMD and UQ II D, we extracted nine questions (three of these items were reversed); five more questions were obtained from UQ III. Adverse effects and pleasantness of the platform were analyzed separately based on individual items of the questionnaire. For more information please see the Supplementary Material.

**TABLE 2:** Structure of usability questionnaire.

**Usability questionnaire**			
UQ I	Demographics and PC experience	12 items	Demographic information (sex, age, education, etc.), previous experience with PC, video games, and HMD games
UQ II HMD	User experience with HMD platform	16 items	Intelligibility, difficulty, pleasantness, input controls, and comfort associated with HMD platform
UQ II D	User experience with desktop platform	14 items	Intelligibility, difficulty, pleasantness, input controls of desktop platform
UQ III	Comparing platforms	13 items	Direct comparison of the platforms in terms of input controls, intelligibility, preference, enjoyment, and spatial orientation. The participants stated their individual preference in both directions in randomized order (e.g., “Spatial orientation was easier for me when the task was presented on desktop” vs. “Spatial orientation was easier for me when the task was presented in HMD”).

*The table displays the four main parts of the usability questionnaire, descriptions, and the corresponding numbers of items.*

### Materials

The experiment was conducted in a NIMH VR lab which was a 7 m long × 5 m wide × 3.5 m high open space. HTC VIVE was used as the HMD platform, with a display resolution of 1080 × 1200 pixels per eye. The motor activity of the participants was tracked using the HTC VIVE headset and controller. The movement in VE was enabled using teleport on the HTC VIVE controller (trackpad) and also by physically walking around the room (walking was limited by the room parameters). The controller trigger was used for the selection of objects. For the desktop platform, a 24-inch monitor with a display resolution of 1920 × 1080 pixels was used. The participants controlled their movements and pick up/drop actions using the keyboard arrows and a computer mouse.

### Procedure

To compare platform usability and platform influence on measured performance, we used an intra-subject design with a counterbalanced order. The participants performed vSST in two conditions with different levels of immersion according to the platform applied: HMD and desktop. During the experiment, we counterbalanced both the order of the platforms (HMD/desktop) and the two vSST task variants (A/B – sets of the lists to remember) to minimize the practice effect on repeatedly measured performance.

**FIGURE 2:**
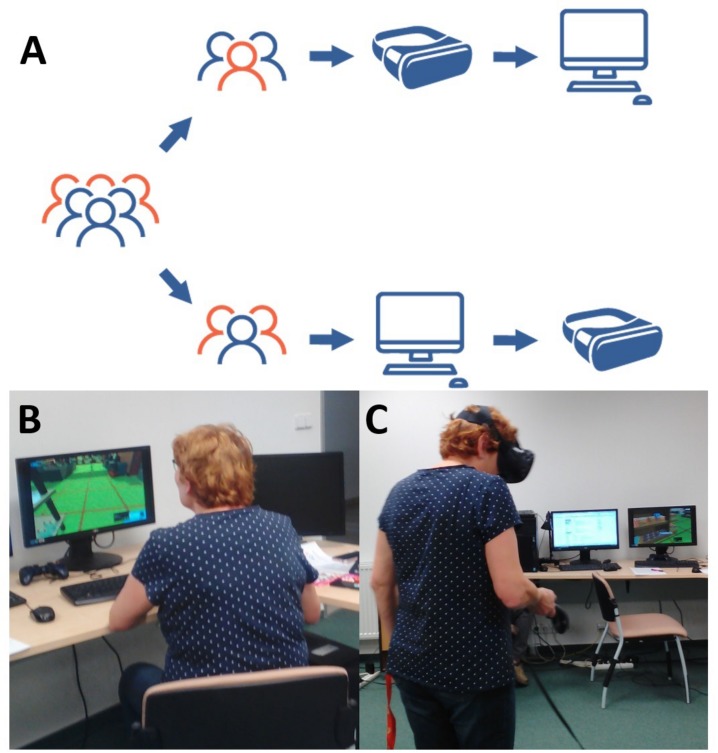
The experimental design of the task Figure **(A)** shows the scheme of intra-subject design with the counterbalanced order of the VR platforms. Figures **(B,C)** show a respondent performing the vSST using desktop **(B)** and HMD platforms **(C)**. The images were obtained with the participant’s consent. The participant signed an informed consent form regarding their publication.

After performing the vSST using the first platform selected according to the counterbalanced order (HMD/desktop, see Figure 2), the participants completed the first two parts of the Usability Questionnaire (UQ I and UQ II HMD/desktop). After performing the vSST using the second platform, participants completed the remaining two parts of the questionnaire (UQ II HMD/desktop and UQ III). Seniors completed a neurocognitive evaluation in a separate session prior to the experiment; young adults were assessed in the end of the experimental procedure.

### Statistics Analysis

The statistical analysis was performed using statistical software IBM SPSS Statistics 19. The group differences in the standard cognitive assessment were analyzed by Mann-Whitney *U* test. Analyses of the differences in vSST performances and user experiences in terms of platform, group and order were examined for statistical significance using ANOVA for repeated measures including the Tukey *post hoc* test. The individual vSST errors and individual questions from usability questionnaire were analyzed using and Wilcoxon Sign Test.

## Results

### Results of the Cognitive Evaluation

In order to compare both tested groups in terms of cognitive functioning controlled by the age effect, prior to the statistical analysis, the raw data acquired from the standard neuropsychological methods were transformed to percentiles according to the Czech normative data (Preiss et al., 2012). We used non-parametric Mann-Whitney *U* test to compare the two groups (seniors and young adults). The normative cognitive performance of seniors in RAVLT and TMT did not differ from that of young adults. The evaluated variables and statistical data for the group comparison can be found in Table 3.

**TABLE 3:** Results of the cognitive assessment.

		**Group of seniors (*N* = 36)**	**Group of young adults (*N* = 25)**	**Seniors vs. Young**	
		***Mean score (SD)***		**Mann-Whitney U**	***p***
**Rey auditory verbal learning test (RAVLT)**					
RAVLT (I-V)	*Words recalled*	51.06⁢(6.89)	56.96⁢(9.74)		
	*percentile*	58.06⁢(22.43)	46.24⁢(26.15)	271.5	0.114
RAVLT delayed	*Words recalled*	11.15⁢(2.5)	12.08⁢(2.44)		
	*percentile*	56.44⁢(23.6)	49.84⁢(29.37)	305.5	0.322
**Trail making test (TMT)**					
TMT-A	*Time (seconds)*	36.77⁢(14.16)	26.2⁢(9.88)		
	*Percentile*	40.76⁢(28.94)	55.2⁢(30.39)	308.5	0.073
TMT-B	*Time (seconds)*	84.99⁢(26.28)	62.88⁢(29.73)		
	*Percentile*	54.63⁢(27.16)	52.36⁢(32.88)	386.5	0.682

*Raw scores and percentiles of test variables (presented in means and SD) reported separately for each tested group and results of statistical comparison between senior and young adult groups. RAVLT, rey auditory verbal learning task; RAVLT I-V, total number of recalled words (highest possible score is 75); RAVLT delayed, number of words recalled after a 30-min delay (from a total of 15 words); TMT-A, trail making test part A; TMT-B, trail making test part B.*

### The Virtual Supermarket Shopping Task Performance

In vSST, we were mainly interested in the number of errors as a parameter measuring the recall accuracy crucial for assessing memory abilities.

#### Cumulative vSST Errors

**FIGURE 3:**
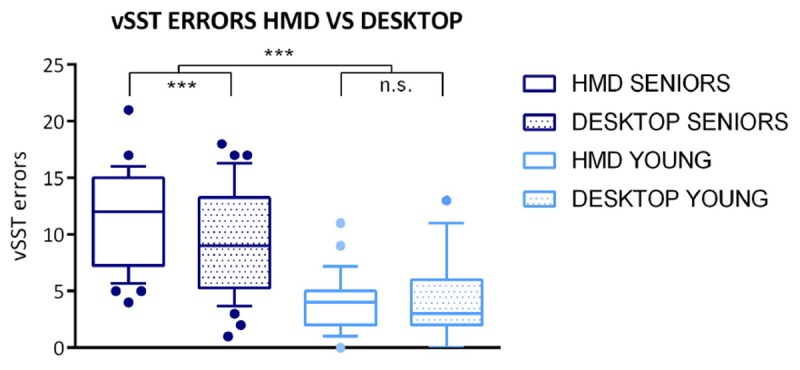
Boxplot for cumulative vSST errors (group/platform). The vSST errors are presented separately for specific age groups and according to the used platform. Boxplots represent the following information: the line is plotted at the median, the box extends from the 25th to 75th percentiles, the whiskers are drawn up/down to the 10th and 90th percentile, and points represent the outliers. The results of statistical analysis are visualized as follows: full line markers represent the group effect and group^*^platform interaction; significance levels are presented as ^∗∗∗^ *p*-value < 0.001; n.s., *p*-value > 0.05.

**FIGURE 4:**
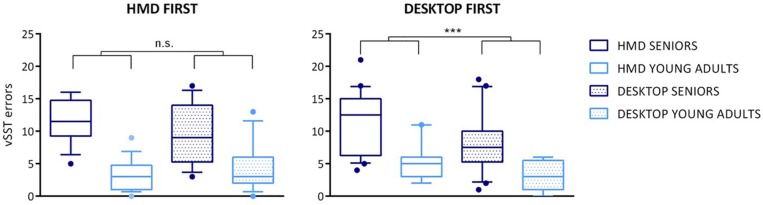
Boxplot for cumulative vSST errors (group/platform/order). The vSST errors are presented for specific age groups and according to the platform. The platform order is displayed by separate graphs. Boxplots represent the following information – the line is plotted at the median, the box extends from the 25th to 75th percentiles, the whiskers are drawn up/down to the 10th and 90th percentile, and points represent the outliers. The results of statistical analysis are visualized as follows: full line markers represent the platform ^*^order interaction effect presented separately for each platform order; significance levels are presented as ^∗∗∗^ *p*-value < 0.001; n.s., *p*-value > 0.05.

In the statistical comparison, we analyzed cumulative errors consisting of combined omission and intrusion errors made during three levels of task difficulty (for five, seven, and nine items on the list). We used a general linear model (GLM) with ANOVA for repeated measures with *platform*, *group*, and *order of platforms* as within-subject factors to analyze vSST errors (see Figures 3, 4). The analysis revealed the main effect of *platform* – the difference between the mean of HMD errors 8.31 (SD = 5.21) and the mean of desktop errors 6.98 (SD = 4.88) is significant, *F*(1,57) = 7.474, *p* = 0.008. A significant main effect was found also in terms of *group* (*F*(1,57) = 45.814, *p* < 0.001) with the mean of errors 20.5 (SD = 8.03) for seniors and the mean of errors 7.8 (SD = 5.02) for young adults. Furthermore, the GLM analysis revealed two interaction effects, for platform^*^group F(1,57) = 4.219, *p* = 0.045 and for platform^*^order F(1,57) = 6.091, *p* = 0.017.

The Tukey *post hoc* test was used to test these interactions, which revealed a significant difference between the HMD errors (mean 11.43, SD = 4.23) and desktop errors in seniors (mean 9.08, SD = 4.64), *p* = 0.001. The performance of the group of young adults did not differ across the platforms (*p* = 0.998). Furthermore, a *post hoc* test showed the difference between HMD errors (mean 9.34, SD = 5.17) and desktop errors (mean 6.69, SD = 4.68) while performing HMD second (platform^*^order), *p* < 0.001, whereas the vSST errors did not differ across the platforms when applying HMD first (*p* = 0.997). No effect of platform order was found with the desktop platform.

#### vSST Errors in Individual Trials

Using the Wilcoxon signed rank test, we analyzed particular vSST errors in individual trials for each tested group to further investigate the variance between the platforms. After applying Bonferroni correction for repeated statistical tests, the difference between the two platforms was not significant in terms of individual vSST errors. Table 4 shows the specific values for each platform and group with appropriate statistics.

**TABLE 4:** Number of errors in individual trials of vSST for each platform and group.

**Number of errors for each vSST trial**						
			**Mean (SD)**		**Wilcoxon sign test**	
**Group**	**Trial**	**Type of error**	**HMD**	**Desktop**	**Z**	***p***
Group of seniors	5 items	Intrusion errors	0.33⁢(0.53)	0.28⁢(0.88)		
		Omission errors	1.39⁢(1.15)	1.22⁢(1.26)		
		Total errors	1.72⁢(1.42)	1.5⁢(1.78)	–1.28	0.199
	7 items	Intrusion errors	1.06⁢(1.09)	0.5⁢(0.91)		
		Omission errors	2.67⁢(1.69)	2.31⁢(1.81)		
		Total errors	3.72⁢(2.33)	2.81⁢(2.16)	–1.88	0.059
	9 items	Intrusion errors	1.47⁢(1.29)	1.23⁢(1.78)		
		Omission errors	4.5⁢(1.36)	3.69⁢(1.69)		
		Total errors	5.97⁢(2.15)	4.91⁢(2.83)	–2.2	0.027
Group of young adults	5 items	Intrusion errors	0.12⁢(0.33)	0.12⁢(0.33)		
		Omission errors	0.44⁢(0.71)	0.28⁢(0.45)		
		Total errors	0.56⁢(0.96)	0.4⁢(0.7)	–0.67	0.499
	7 items	Intrusion errors	0.12⁢(0.33)	0.28⁢(0.67)		
		Omission errors	0.56⁢(0.82)	0.64⁢(1.03)		
		Total errors	0.68⁢(0.9)	0.92⁢(1.57)	–0.32	0.749
	9 items	Intrusion errors	0.68⁢(0.9)	0.68⁢(0.98)		
		Omission errors	1.92⁢(1.28)	1.96⁢(1.42)		
		Total errors	2.6⁢(1.7)	2.64⁢(1.75)	–0.08	0.929

*The table reports mean number and SD of total errors, intrusions and omissions, and statistical difference in total errors for each group according to the platform used. The differences for total errors obtained in individual trials are reported with corresponding statistics. There are no significant effects after applying the Bonferroni correction (α = 0.017).*

### Usability Questionnaire

#### Cumulative Score

**FIGURE 5:**
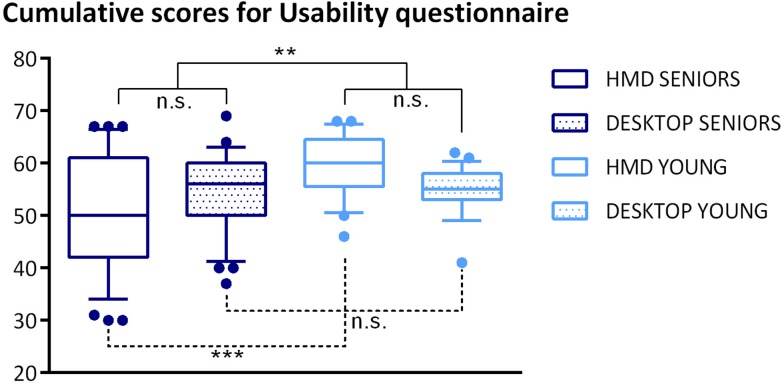
Boxplots of cumulative scores of the Usability questionnaire. Boxplots represent the following information – the line is plotted at the median, box extends from the 25th to 75th percentiles, the whiskers are drawn up/down to the 10th and 90th percentile, and points represent the outliers. The results of statistical analysis are visualized as follows: full line markers represent the group effect, dashed line markers represent group^*^platform interaction effects, significance levels are presented as ^∗∗∗^ *p*-value < 0.001; ^∗∗^ *p*-value < 0.01; n.s., *p-*value > 0.05.

We applied a general linear model (GLM) with ANOVA for repeated measures with *platform*, *group*, and *order of platforms* as within-subject factors to analyze the summary results for the usability of individual platforms (for details, see Figure 5).

The analysis revealed a main effect of *group* with the mean usability score 105.29 (SD = 11.71) for seniors and 114.64 (SD = 6.40) for young adults [*F*(1,56) = 10.986, *p* = 0.002]. Furthermore, the analysis revealed only one interaction effect for platform^*^group F(1,56) = 6.148, *p* = 0.016.

For further analysis of this interaction effect, we used the Tukey *post hoc* test, which revealed a significant difference (*p* < 0.001) between HMD scores in seniors (mean 50.49, SD = 11.29) and HMD scores in young adults (mean 59.72, SD = 5.86); the user experience with the desktop platform showed no group effect (*p* = 0.999). There was no significant difference between the platforms’ usability scores in either of the age groups.

#### Individual Questions

In addition to cumulative scores calculated for individual platforms and groups, we analyzed the results for individual items from sections UQ II HMD and UQ II D. Because of the Likert scale usage, we investigated the difference between the platforms with a non-parametric Wilcoxon-signed rank test. After Bonferroni correction for repeated statistical comparison (α = 0.01), we observed a significant difference between the platforms only in the group of young adults. Specifically, the young adults preferred HMD (mean 4.2, SD = 1.11) over the desktop platform (mean 2.04, SD = 0.97), *Z* = −3.42, *p* < 0.001. The young adults also enjoyed the HMD (mean = 4.32, SD = 0.9) significantly more than the desktop (mean 2, SD = 0.81), *Z* = −3.98, *p* < 0.001). For details, see Table 5.

**TABLE 5:** Mean score of individual questions.

**Mean score for individual questions for each platform**					
**Mean (SD)**				**Wilcoxon sign test**	
		**HMD**	**Desktop**	**Z**	***p***
Group of seniors	Intelligibility	2.71⁢(1.34)	3.45⁢(1.35)	–1.66	0.097
	Preference	3.11⁢(1.52)	3.02⁢(1.46)	–0.07	0.948
	Spatial orientation	2.94⁢(1.53)	3.42⁢(1.28)	–1.3	0.195
	Input controls	2.91⁢(1.44)	3.65⁢(1.25)	–1.77	0.077
	Enjoyment	3.11⁢(1.36)	3.14⁢(1.28)	–0.16	0.874
Group of young adults	Intelligibility	2.6⁢(1.11)	2.36⁢(1.11)	–0.83	0.408
	Preference	4.2⁢(1.11)	2.04⁢(0.97)	–3.42	0.001^*^
	Spatial orientation	3.28⁢(1.2)	2.88⁢(1.01)	–1.01	0.315
	Input controls	3.16⁢(1.43)	3.2⁢(1.11)	–0.09	0.926
	Enjoyment	4.32⁢(0.9)	2 (0.81)	–3.98	$<0.001^{*}$

We report mean scores (SD) for individual statements from the usability questionnaire for each platform and group separately. The difference is reported with corresponding statistics. Significant effects after applying the Bonferroni correction (α = 0.01) are marked with a symbol ^*^.

#### Side Effects

In the usability questionnaire sections UQ II HMD and UQ II D, we asked participants about the adverse effects of the specific platform. The participants were asked about unpleasant feelings connected with the task; if they reported the presence of unpleasant feelings, they were asked to specify the feeling *(Was the unpleasant feeling connected with experienced discomfort? Select one or more options from the list of the possible adverse effects…).* The incidence of the side effects, including their specific characteristics, are reported in Table 6. Importantly, the reported side effects were small and no participant asked to terminate their participation in the study.

**TABLE 6:** The incidence of reported side effects associated with VR experience.

***Group***	***HMD***	***Desktop***
*The group of seniors*	Six (17%) of the participants reported “feeling sick” with the HMD platform. Specifically, four seniors felt disoriented, three felt nauseous, three felt dizzy, two experienced headaches, two experienced dry eyes or eye fatigue while using HMD.	One senior (3%) reported “feeling sick” with the desktop platform. Specifically, the participant reported experiencing headache during the experiment.
*The group of young adults*	None of the participants reported unpleasant feelings connected with the usage of HMD.	None of the participants reported “feeling sick” while completing the vSST on desktop.

## Discussion

The main findings of the presented study are the significant age-related differences across the tested VR platforms (HMD vs. desktop) that were identified not only in terms of assessed performance but also in user experience. This age-related effect is not surprising as the addressed groups typically differ in experience with new technologies, of which HMD is an example.

### Memory Recall

The study aimed to evaluate possible effects of immersion level (desktop vs. HMD platform) on the ability to recall items from a presented shopping list (participant accuracy was expressed as the number of errors in the vSST task). According to our results, the seniors made significantly more errors when using the HMD platform than when using the desktop platform. The vSST recall performance of the young adults was stable regardless of the platform used. Our findings for the senior group are in accordance with some previous studies investigating navigation and spatial memory (Sousa Santos et al., 2009) that associated the desktop platform with superior performance. Similar findings were reported in a study by Mania and Chalmers (2001) that investigated the ability to recall information from a seminar presented in four conditions: a real-world environment, desktop, HMD, and audio-only. According to that study, the memory performance was the best in the real-world scenario and the worst in the HMD platform. Moreover, the memory recall was statistically higher in the desktop platform than in HMD.

Other studies favor the HMD platform in terms of spatial memory recall (Ruddle et al., 1999; Bowman et al., 2009; Murcia-López and Steed, 2016). A possible explanation for such contradictory results is that the benefits of HMD, such as the active movement control and rotation controlled by head movements, are highlighted in studies that assess spatial navigation abilities. This potential of HMD might be overshadowed by different factors in non-spatial memory tasks.

We speculate that the presentation of the recall tasks in HMD can lead to perceptual or cognitive overload; the participants are present “inside” a virtual environment with possibly higher perceptual stimulation (Richards and Taylor, 2015). The possibility that higher immersion is a distracting factor while learning a task has been investigated. Despite the motivational potential of HMD, the higher immersion can distract participants from the studied material (Moreno and Mayer, 2004; Richards and Taylor, 2015; Parong and Mayer, 2018). Makransky et al. (2019) pointed out a possible effect of higher levels of cognitive load (measured by EEG) associated with more immersive technology. These findings may explain the inferior HMD performance observed in the seniors, considering the goal of the task (remembering a shopping list). The difference between the young adult and senior subjects in our study could be thus related to the lower ability to inhibit distracting information in seniors (Moreno and Mayer, 2004).

On the other hand, the higher stimulation and distraction of the HMD platform might in some way reflect its higher ecological validity in comparison to the desktop platform. For this reason, it would be beneficial to add an extra measure of ecological validity in future comparative studies.

Importantly, most of the mentioned studies did not investigate age-related differences. Such a comparison, in terms of acceptance of new technologies and memory assessment, is important, as memory decline is typical in older adults (Small, 2001). A comparison of the different platforms and two age groups (young adults ages 16–35; seniors ages 60–75) was conducted by Rand et al. (2005). The authors used the “Virtual Office” environment, which was developed to assess attention and memory performance (Rizzo et al., 2002). Based on the obtained results, the performance of both age groups was significantly lower when using the HMD platform. These findings are only partially in accordance with our results as the authors observed an inferior HMD performance also in young adults. This difference in the obtained results could be explained by technological progress in HMD devices in recent years.

Regardless of the observed effect of platform on performance in the memory task in seniors, the fact that the group of seniors performed worse in both platforms than the group of young adults confirms the validity of vSST for memory assessment. The validity of the task was also indicated in previous studies conducted on healthy young adults and patients with chronic schizophrenia (Plechatá, 2017; Plechatá et al., 2017).

By counterbalancing the order of the platforms and task variants applied we controlled for possible effects of fatigue and practice effect. A similar approach was applied in other studies (Ruddle et al., 1999; Sousa Santos et al., 2009). Additionally, in our study the platform order was used as a confounding variable in the presented GLM analysis. We expected that previous experience with the task using the desktop platform would improve consecutive HMD performance. Surprisingly, when using the desktop platform first, the participants from both age groups made higher numbers of errors using HMD than they did using the desktop platform. In contrast, if the HMD platform was presented first, the performance was comparable between both platforms.

Several possible factors might have induced this interaction effect. We argue that the HMD performance might be influenced by the fatigue of the subjects (due to the repeated measurement); the results would differ with the desktop platform, as most of the participants had previous experience with the desktop but not with the HMD platform. Higher sensitivity to fatigue in seniors (Eldadah, 2010) can be also associated with the perceptual overload of HMD, mentioned above, which can lead to higher difficulty of the task itself. Unfortunately, to our knowledge none of the previous studies analyzed the effect of the order in which the platforms were applied (Ruddle et al., 1999; Sousa Santos et al., 2009).

### User Experience

According to the results of the usability questionnaire, the user experience with HMD or desktop platforms is not comparable across the different age groups. The seniors evaluated the HMD experience differently than the young adult subjects. In general, the young adults evaluated the experience with higher scores than the seniors did. However, in the cumulative score of the questionnaire, we found no significant preference for HMD or desktop platform in the young adult or senior participants. The fact that the young adults scored higher in the usability questionnaire than seniors did regardless of the platform may reflect a difference in their attitude toward the specific task or toward computer technology in general.

In respect to individual categories evaluated in the usability questionnaire, the participants in our study favored neither HMD nor desktop platforms in terms of input controls or intelligibility of the task. Nevertheless, the younger adults stated that they liked the HMD platform more than desktop platform. Similarly, the younger participants enjoyed the experience of using HMD more than using the desktop platform. Our findings are in line with the results of previous studies that favored the HMD platform over desktop and screen platforms (Adamo-Villani and Wilbur, 2008; Sousa Santos et al., 2009) in cognitive assessments of young adults. The participants of these studies preferred HMD in general; they considered it more intuitive (Sousa Santos et al., 2009) and more fun (Adamo-Villani and Wilbur, 2008). As both evaluated factors are closely related to motivation, these results might also be supported by studies focusing on the potential of HMD for educational purposes showing that the more immersive technology increased motivation to study (Moreno and Mayer, 2004; Richards and Taylor, 2015; Parong and Mayer, 2018).

On the other hand, the user experience evaluated by seniors in our study did not reflect these findings as the seniors preferred neither HMD nor the desktop platform. Unfortunately, to our knowledge, the existing studies comparing the two platforms in cognitive assessments did not involve older adults. The only exception is the study by Rand et al. (2005), which did not investigate the platform-dependent difference in the user experience. None of the seniors recruited in our study had previous experience with HMD and virtual reality games, while most of the seniors were experienced with computers. As was demonstrated previously, repeated exposure to immersive VR can lead to a decrease of its adverse effects (Taylor et al., 2011); therefore, it could be expected that it also leads to the improvement in other variables of the user experience. The role of repeated exposure either to HMD or to the task itself should be further studied in order to evaluate its potential for cognitive training and remediation.

Considering the adverse effects of immersive virtual reality, the presence of typical side effects associated with HMD were very low among seniors. Moreover, no cybersickness symptoms were reported in the group of young adults. The higher acceptance of immersive VR in this study without negative side effects could be associated with the design and navigation system used in the task (combination of teleport and active movement).

### Limitations

Despite our effort to control for other confounding factors (e.g., by a counterbalanced order of the platforms), we admit that the differences observed in the task performance could have been influenced by other variables.

In particular, the inferior performance in HMD observed in the group of seniors could be associated with the small but important distinction of the experimental procedure. In contrast to the desktop platform, during the HMD condition the participant was instructed first to take off the HMD and then to sit at a nearby table and play a visuospatial game LEU (used as a distractor in both platforms). Thus, with the HMD platform, there was a specific additional distractor in the form of removing the HMD glasses. Moreover, the participants were standing during HMD and sitting while using desktop platform. The different motor involvement in the task and different control system could influence task performance. This effect could be even stronger in a group of seniors with lower visuospatial coordination abilities (Hoogendam et al., 2014). In future studies, the distinction in the experimental setting could be eliminated by adding a distraction task directly into the VR application, thus not requiring participants to take off HMD glasses during the procedure.

Despite the investigation of the role of immersion, we did not study the sense of presence that is typically measured by questionnaires (Slater et al., 1994) after performing the VR task. As the level of presence was not a key variable in this study, it was not investigated mainly due to higher time demands of the experimental procedure in individual participants. It could be, however, beneficial to study the difference in the sense of presence especially in seniors, as it might explain the age-related variance in the platform performance and user experience in more detail. It was previously shown that the sense of presence is typically higher when using more immersive technology (Slater, 2018). A recent study (Corriveau Lecavalier et al., 2018) showed that both young and older adults experience comparable level of presence in immersive VR environment. However, this study also reports positive correlation between the performances measured in a Virtual Shop task aimed at episodic memory and reported sense of presence in seniors. These results do not explain the negative effect of higher immersion on performance of seniors found in our study. This discrepancy should be therefore addressed in future studies.

Finally, despite the reasonable number of participants recruited in this study, the number of subjects with limited or no PC experience made it impossible to evaluate the possible benefits of HMD technology in such participants, especially in the group of seniors. Future studies should investigate the role of ecological validity in terms of VR immersion level and behavioral outcomes of the participants.

## Conclusion

In the presented study, we studied the age-related differences between HMD and desktop platforms in memory assessment using an intra-subject design. Groups of seniors and young adults performed a virtual Supermarket Shopping task aimed at episodic memory using HMD and desktop platforms in a counterbalanced order. We focused on the role of the level of immersion on the task performance and its usability. According to our results, the senior performances were inferior in HMD in contrast to the desktop platform. The measured performance of the young adults was stable and comparable regardless of the platform used. In the context of the diagnostic application of VR tasks in seniors, our results indicate that it is necessary to create separate normative data for the task, dependent on the VR platform used for the assessment. Furthermore, the HMD platform was more influenced by fatigue of the participants, as the performance was lower on HMD for both groups when performing HMD as the second platform. In general, the seniors evaluated their user experience lower than the young adults did regardless of the platform used. We did not find any significant platform-related differences in overall user experience in any of the tested groups. However, according to the data obtained in individual items of the questionnaire, the young adults tended to prefer HMD over the desktop platform.

Our results indicate that performing the task with HMD may be more difficult than with the desktop platform; this difficulty may be associated with perceptual overload in the senior subjects. It might also indicate the superior ecological validity of the HMD presented task; this possibility should be studied further. The fact that the user experience did not differ across the platforms used and only minimal side effects were reported indicate that highly immersive technology may be well accepted by aging adults. This may have implications for the further use of HMD in cognitive remediation; this has been proposed in previous studies (Gamito et al., 2014). We hypothesize that with repeated HMD experiences, seniors will find it more motivating and intuitive to use than the desktop platform. However, in the context of diagnostic use of VR in a single session, the benefits of higher immersion are questionable.

## Ethics Statement

This study was carried out in accordance with the recommendations of “NIMH CZ Ethics Committee” with written informed consent from all subjects. All subjects gave written informed consent in accordance with the Declaration of Helsinki. The protocol was approved by the “NIMH CZ Ethics Committee.”

## Author Contributions

AP was responsible for the design of the experiment and data collection. VS developed the virtual supermarket shopping task. DF was responsible for recruiting the participants. IF supervised the whole study and together with AP was responsible for writing the manuscript.

## Conflict of Interest Statement

The authors declare that the research was conducted in the absence of any commercial or financial relationships that could be construed as a potential conflict of interest.

## Footnotes

1 https://unity3d.com/

2 http://www.leubrainstimulator.com/
