# Supplementary material for: Age-Related Differences With Immersive and Non-immersive Virtual Reality in Memory Assessment
Source: Front Psychol. 2019 Jun 11;10:1330. doi: 10.3389/fpsyg.2019.01330 (PMC6579887; doi:10.3389/fpsyg.2019.01330)
Supplement: Supplementary file 1 [file Data_Sheet_1.docx]

***Supplementary Material***

**Usability questionnaire**

Please note:

The information written in italics clarifies the form of the questionnaire. It was not included in the final questionnaire. The questions are written in bold.

# The question was included in HMD platform cumulative score.

$ The question was included in desktop platform cumulative score.

* The questions marked with an asterisk were answered on 5-point scale with extreme values of Definitely not (1) and Definitely yes (5)

**UQ part I. Demographics and PC Experience**

**State your age:** *(open answer specified as a number)*

**State the length of your education in years:** *(open answer specified as a number)*

**State your highest level of educational attainment:** *(multiple-choice question)*

- vocational school
- high school
- university degree

**State your sex:** *(multiple-choice question)*

- male
- female

**State your field of employment (if you are retired, please state your previous employment):** *(open answer)*

**Are you retired?** *(multiple-choice question)*

- yes
- no

**How often do you use a computer?** *(multiple-choice question)*

- never
- once per month
- a few times per month
- once per week
- a few times per week
- daily

**I am used to using a computer.***

**I have experience playing video games.***

**I have experience playing games in virtual reality.***

**I am familiar with HTC VIVE glasses.***

**UQ part II. User Experience with HMD platform**

**# The task was clear to me in all its parts.***

**# I understood all the verbal instructions.***

**# The task was easy to control.***

**# During the testing, at some point I didn't know what to do or how to do something.***

- *If the answer was yes or definitely yes (4/5 points), a follow-up question appeared:*
- **When did this happen?** *(open answer)*

**# The task seemed complicated or even unmanageable for me.***

**# During the testing, I felt uncomfortable.***

- *If the answer was yes or definitely yes (4/5 points), a follow-up question appeared:*
  - **This feeling was linked to the fact that I did not know how to correctly proceed in the task**.*
  - **This feeling was linked to a feeling of being sick.***
  - **Did you experience any of these symptoms?** *(multiple choice question)*
    - nausea
    - vertigo
    - eye strain/sore eyes
    - headache
    - disorientation

**# The environment seemed natural to me.***

**# I was able to navigate properly in the virtual space.***

**# I recognized all the goods in the supermarket.***

**Would you change anything about the task? Please specify:** *(open answer)*

**I was uncomfortable using virtual glasses.***

**Did you take off the virtual glasses during testing? If so, how many times? (If you did not take off the glasses, type 0)** *(open answer specified as a number)*

**UQ part II. User Experience with Desktop platform**

**$ The task was clear to me in all its parts.***

**$ I understood all the verbal instructions.***

**$ The task was easy to control.***

**$ During the testing, at some point I didn't know what to do or how to do something.***

- *If the answer was yes or definitely yes (4/5 points), a follow-up question appeared:*
- **When did this happen?** *(open answer)*

**$ The task seemed complicated or even unmanageable for me.***

**$ During the testing, I felt uncomfortable.***

- *If the answer was yes or definitely yes (4/5 points), a follow-up question appeared:*
  - **This feeling was linked to the fact that I did not know how to correctly proceed in the task**.*
  - **This feeling was linked to a feeling of being sick.***
  - **Did you experience any of these symptoms?** *(multiple choice question)*
    - nausea
    - vertigo
    - eye strain/sore eyes
    - headache
    - disorientation

**$ The environment seemed natural to me.***

**$ I was able to navigate properly in the virtual space.***

**$ I recognized all the goods in the supermarket.***

**Would you change anything about the task? Please specify:** *(open answer)*

**UQ part III. Comparing Platforms**

**$ The task was easier to comprehend on the computer monitor (compared to the virtual glasses).***

**$ The task was easier to control on the computer monitor (compared to the virtual glasses). ***

**$ I liked the task on the computer monitor more (compared to the virtual glasses).***

**# The task was easier to comprehend with virtual glasses (compared to the monitor).***

**# I liked the task with the virtual glasses more (compared to the monitor).***

**# Spatial orientation was easier with the virtual glasses (compared to the monitor).***

**$ I enjoyed the task on the computer monitor more (compared to the virtual glasses). ***

**# The task was easier to control with the virtual glasses (compared to the monitor).***

**The task was more pleasant with the virtual glasses (compared to the monitor).***

**# I enjoyed the task with virtual glasses more (compared to the monitor).***

**$ Spatial orientation was easier on the computer monitor (compared to the monitor).***

**The task was more pleasant on the computer monitor (compared to the monitor).***

**What approach did you choose to memorize the items on the list (on both platforms)?** *(multiple-choice question)*

- 1. memorizing (repeating in the head)
  2. sorting items into categories (fruits, vegetables, cosmetics ...)
  3. visualizing the items in the mind
  4. association between items (e.g. spoon belongs on the plate)
  5. visualization of the route or deployment of items in the supermarket
  6. creating a story from the items on the list
  7. other answer (please specify):
